# The Changing Characteristics of Technologies Covered by Medicare’s New Technology Add-on Payment Program

**DOI:** 10.1001/jamanetworkopen.2020.12569

**Published:** 2020-08-27

**Authors:** Christopher R. Manz, Justin E. Bekelman, Jalpa A. Doshi

**Affiliations:** 1 Department of Medicine, Division of Hematology and Oncology, University of Pennsylvania, Philadelphia; 2 Departments of Radiation Oncology and Medical Ethics and Health Policy, University of Pennsylvania; 3 Department of Medicine, Perelman School of Medicine, University of Pennsylvania

## Abstract

This economic evaluation explores how Medicare’s new technology add-on payment program (NTAP), which provides reimbursement for newly available drugs and devices, has changed during its lifetime since its initiation in 2013.

## Introduction

Medicare’s inpatient prospective payment system pays for inpatient hospitalizations according to diagnosis related groups (DRGs), with reimbursement based on average costs for each DRG in the previous 2 years. Because this calculation cannot incorporate payments for newly available expensive drugs or devices provided during hospitalizations, Medicare has established the new technology add-on payment (NTAP) program, which provides additional reimbursement of up to 50% (65% starting fiscal year 2020) of the cost of NTAP-approved technologies that are new, provide “substantial clinical benefit,” and exceed cost thresholds for the applicable DRGs.^1^ NTAP payments are intended as bridge payments for the 2 to 3 years it takes a DRG to recalibrate.^1^ We examined how NTAP technology characteristics have changed during NTAP’s 18-year existence.

## Methods

Data on NTAP technologies, cost, and payments were extracted from publicly reported data in Medicare’s annual inpatient prospective payment system final rules for fiscal years 2003-2020.^2^ Potential hospital loss was calculated as the annual final rule–reported technology cost minus the maximum NTAP payment (eMethods in the Supplement).

## Results

The number of technologies covered by NTAP each year has increased, peaking at 18 technologies for fiscal year 2020 (Figure). Comparison of the first and second 9-year periods of NTAP (fiscal years 2003-2011 vs 2012-2020) shows that the number of unique NTAP technologies increased 3.7-fold, from 10 to 37 (Table). The proportion of technologies that were drugs (vs devices) increased from 10% (n = 1) to 59% (n = 22); that were oncology-related, from 10% (n = 1) to 32% (n = 12); and that resulted in potential hospital losses of greater than $30 000, from 10% (n = 1) to 24% (n = 9) across the 2 periods. Despite the increased 65% reimbursement, 6 of the 18 fiscal year 2020 technologies may incur potential hospital losses of greater than $10 000, including 3 greater than $50 000. The median oncology technology cost for all years was $74 725 vs $12 291 for nononcology technologies. Median costs were lower for drugs than devices ($11 312 vs $20 265), but high-priced drugs were more highly skewed (maximum cost, $382 605 vs $168 761).

**Figure.**
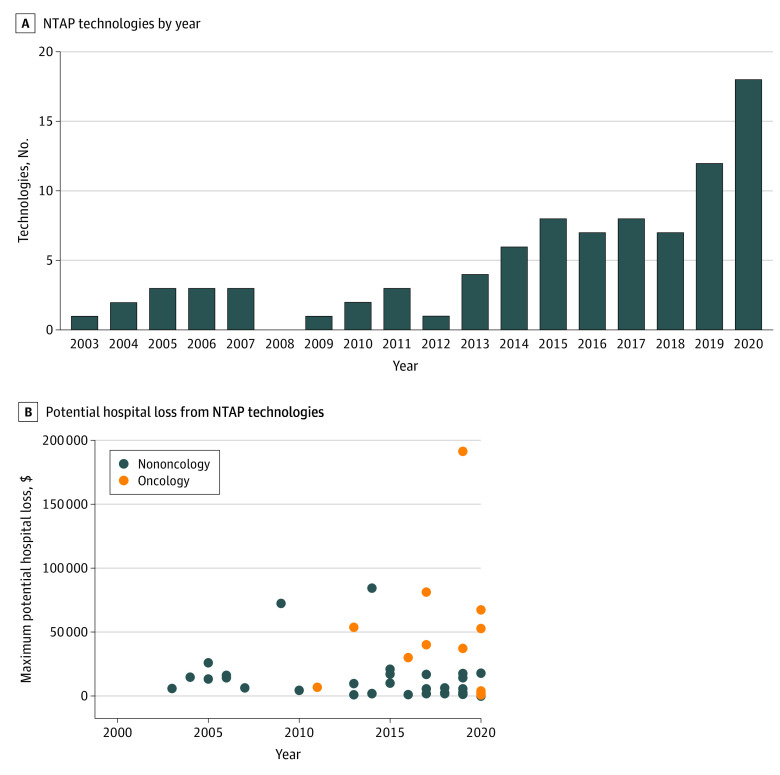
New Technology Add-on Payment Technology (NTAP) Trends by Fiscal Year A, NTAP technologies covered in each fiscal year (fiscal years 2003-2020). An individual technology is included in each year for which it is NTAP approved (up to 3 years). B, Potential hospital loss from NTAP technologies, based on first fiscal year of NTAP approval. Fiscal year 2020 hospital losses calculated with the newly revised maximum NTAP rate of 65% (75% for 1 qualified infectious disease product). All other years calculated with an NTAP rate of 50%.

**Table.** New Technology Add-on Payment Technology Characteristics, Fiscal Years 2003-2011 vs 2012-2020

Characteristic	FY 2003-2011	FY 2012-2020
Unique new technologies	10	37
No. of technologies covered per year, mean^a^	2	7.9
Therapeutic areas with approved technologies	7	12
Oncology vs nononcology technologies, No. (%)^b^	Oncology: 1 (10)	Oncology: 12 (32)
	Cardiovascular: 3 (30)	Cardiovascular: 8 (22)
	Infectious disease: 0	Infectious disease: 5 (14)
	Hematology: 0	Hematology: 4 (11)
	Other: 6 (60)	Other: 8 (22)
Technology type, No. (%)	IV medications: 1 (10)	IV medications: 16 (43)
		Devices:13 (35)
		Oral medications: 5 (14)
	Devices: 9 (90)	Blood product: 1 (3)
		Nasal spray medication: 1 (3)
		Diagnostic test: 1 (3)
Technologies with potential hospital loss greater than $30 000, No. (%)	1 cardiovascular device (10)	8 oncology drugs; 1 ophthalmology device (24)
Technology cost, median (IQR), $	Oncology: 74 725 (11 250-151 000)	
	Nononcology: 12 291 (3753-32 552)	
	Drug: 11 312 (3862-77 571)	
	Device: 20 265 (9106-34 250)	

Abbreviations: FY, fiscal year; IQR, interquartile range; IV, intravenous; NTAP, new technology add-on payments.

^a^ An individual technology may be approved for NTAP for up to 3 years.

^b^ Other includes orthopedics, neurology, pulmonology, shock, anesthesia, gastroenterology, ophthalmology, psychiatry, and urology.

## Discussion

This study demonstrates that the number, types, and costs of NTAP technologies have changed significantly since the program’s inception, driven particularly by oncology drugs. Administration routes have diversified. For fiscal year 2020, oral cancer drugs erdafitinib and apalutamide received NTAP approval essentially as expensive home medications that patients should continue to receive during hospitalizations but are not covered by DRG reimbursement. Many new oral medications fit these criteria, possibly opening the floodgates for similar NTAP approvals. Yet when NTAP approval expires for these drugs, the dozens of underlying DRGs still will not meaningfully reflect these medication costs. Device approvals may also increase because of recent policy changes loosening NTAP criteria for Food and Drug Administration–designated “breakthrough devices.”^2^

Very high prices for some products have increased the possibility of high hospital losses, given current maximum NTAP payments. Our loss calculation has limitations because hospitals may negotiate lower prices or recoup some losses through outlier payments; however, they also may not qualify for maximum NTAP payments. Nevertheless, these data suggest that an analysis of actual hospital expenditures and reimbursement is warranted to assess the adequacy of current payment policy and whether NTAP provides effective bridge payments to higher DRG reimbursement.

A recent study raised concerns that many NTAP technologies may not provide substantial clinical benefit.^3^ Drawing from other countries’ experiences, others highlighted the need for broad US policy reform that aligns technology prices with value.^4^ Our study adds to this ongoing discussion by raising questions regarding adequacy of reimbursement of these technologies and whether, despite recent increases, NTAP payments will be sufficient to incentivize hospitals to offer these therapies to patients in need. Further research and policy improvements are needed to ensure patient access to high-value technologies.

## Notes

Supplement. **eMethods.**
