# Supplementary material for: The Changing Characteristics of Technologies Covered by Medicare’s New Technology Add-on Payment Program
Source: JAMA Netw Open. 2020 Aug 27;3(8):e2012569. doi: 10.1001/jamanetworkopen.2020.12569 (PMC7453306; doi:10.1001/jamanetworkopen.2020.12569)
Supplement: Supplement. — eMethods. [file jamanetwopen-3-e2012569-s001.pdf]

Supplementary Online Content

Manz CR, Bekelman JE, Doshi JA. The changing characteristics of technologies covered by Medicare’s new technology add-on payment program. *JAMA Netw Open*. 2020;3(8):e2012569. doi:10.1001/jamanetworkopen.2020.12569

eMethods.

This supplementary material has been provided by the authors to give readers additional information about their work.

eMethods

IRB exemption. This study used publicly reported data from the CMS website and did not require Institutional Review Board approval.

Calculation of potential hospital loss. In the annual Inpatient Prospective Payment System (IPPS) Final Rule published every August, Medicare reports the cost of each NTAP technology that is used for the basis of the maximum NTAP payment calculation. Typically, this cost is the list price of the technology. Medicare then reports the maximum NTAP payment for that financial year, which is 50% of the NTAP technology cost for FY2003-2019 and 65% for FY2020 (75% for qualified infectious disease products in FY2020). The potential hospital loss was calculated by subtracting the maximum NTAP payment of the technology from the Medicare-reported cost of the technology. All costs were adjusted to 2019 dollars using the medical component of the Consumer Price Index.

A few caveats with our calculation of potential hospital losses should be noted. It is possible that hospitals may not qualify for maximum NTAP payments and hence face even higher losses than we have estimated. The annual IPPS Final Rule sets the maximum NTAP payment that a hospital may receive for an individual claim, but relies on a complex formula involving hospital-specific payment characteristics (such as the hospital's cost-to-charge ratio) and individual patient claims for the entire hospitalization during which the NTAP technology was administered (based on hospital charges, not actual cost to the hospital) to determine the actual NTAP payment that a hospital receives for each claim. Thus, if the Medicare payment formula yields a sum that is less

© 2020 Manz CR, et al. *JAMA Network Open.*

than the maximum allowable NTAP reimbursement, the hospital will receive the lesser amount. In a simplified example, if a hospital has a cost to charge ratio of 0.25, obtains an NTAP technology at list price and marks up the charge for the NTAP technology less than 400%, the hospital may not qualify for the full NTAP payment.

On the other hand, hospitals may negotiate lower technology prices with the manufacturers or recoup some losses through outlier payments and hence face lower losses than we have estimated. It should be noted that while hospitals may receive outlier payments, these payments will reduce but not eliminate hospital losses given Medicare's approach to calculating these outlier amounts. In a simplified description, Medicare multiplies hospital charges for a specific inpatient admission by the hospital's cost-to-charge ratio to determine hospital costs. To calculate reimbursement, Medicare multiplies the applicable DRG payment by hospital-specific weighting and adds in NTAP payments, if applicable. If the calculated hospital costs are more than approximately $26,000 (the fixed loss outlier threshold) above the reimbursement, then hospitals may qualify for outlier payments. Outlier payments cover 80% of costs that exceed the fixed-loss outlier threshold of approximately $26,000 above the reimbursement. In a simplified example, if Medicare determines that reimbursement for a specific inpatient admission is $80,000 (inclusive of NTAP payments), the hospital may qualify for an outlier payment if Medicare determines that the hospital's costs, as calculated above exceed the reimbursement plus the fixed loss outlier threshold (i.e. $80,000 + $26,000 = $106,000). The outlier payment would be equal to 80% of the costs above $106,000. In this instance, the hospital loss would be $26,000 plus 20% of the Medicare-determined costs above $106,000.

© 2020 Manz CR, et al. *JAMA Network Open.*

Finally, as with any hospitalization, hospitals may make a profit off of a hospitalization exclusive of NTAP technology-related costs, and such profits may offset losses that the hospital experiences due to under-reimbursement of NTAP technology costs.

For further details about how Medicare calculates payment under the Inpatient Prospective Payment System, see: https://www.cms.gov/Outreach-and-Education/Medicare-Learning-Network-MLN/MLNProducts/Downloads/AcutePaymtSysfctsht.pdf

© 2020 Manz CR, et al. *JAMA Network Open.*
